# Gels as Templates for the Syntheses of Shape-Controlled Nanostructured Materials

**DOI:** 10.3390/gels4010010

**Published:** 2018-01-16

**Authors:** Pablo H. Di Chenna

**Affiliations:** Universidad de Buenos Aires, Consejo Nacional de Investigaciones Científicas y Técnicas, Unidad de Microanálisis y Métodos Físicos Aplicados a la Química Orgánica (UMYMFOR), Departamento de Química Orgánica, Facultad de Ciencias Exactas y Naturales, Pabellón 2, Ciudad Universitaria, Buenos Aires C1428EG, Argentina; dichenna@qo.fcen.uba.ar

**Keywords:** template, nanoparticle, nanomaterial, structure transcription, gel, organogel, hydrogel

The preparation of inorganic, organic and/or hybrid nanostructured materials with controlled shape and size is crucial for the development of nanotechnology, and it is nowadays the focus of intense research. Inorganic materials and, to a lesser extent, organic materials with well-defined morphology or porosity at the nanoscale can be routinely synthesized by the use of different “templates”. In particular, gels—both polymeric and supramolecular—provide a diversity of nanoarchitectures such as tubes, rods, ribbons, tapes, helices, etc. These superstructures have a unique and well defined morphology that can be used as templates for transcribing their structure into a range of inorganic and organic materials with many potential applications. Moreover, in the case of low-molecular-weight gelators (LMWGs), the successful transcription of these structures to a nanomaterial can give direct information on the morphology of the superstructure of the gel in native form, information that is otherwise difficult to obtain in a direct way [1,2]. 

This Special Issue of *Gels* entitled “Gels as Templates for Transcription” presents a clear picture of the variety of approaches for the transcription of LMWG superstructures into inorganic and organic polymeric materials and is comprised of a review and three original articles. Susuki et al. [3] report on the preparation of titanium dioxide nanotubes and hybrid nanotubes prepared with various metal oxides such as Ta_2_O_5_, Nb_2_O_5_, ZrO_2_, and SiO_2_ by the sol–gel polymerization using a simple l-lysine-based LMWG, demonstrating that the composition ratio of hybrid nanotubes is controllable by the feed ratio of raw materials. A novel and simple transcription strategy for the template-synthesis of CePO_4_·xH_2_O nanofibers was reported by Llusar et al. [4]. The strategy allowed the preparation of nanofibers with improved morphology using a pH-sensitive hydrogel based on a glycine-alanine lipodipeptide as a structure-directing scaffold. The phosphorylated hydrogel was employed as a template to direct the mineralization of a high-aspect-ratio nanofibrous cerium phosphate, which was in situ formed by the diffusion of aqueous CeCl_3_ and subsequent drying and annealing treatments. The nanofibrous CePO_4_ exhibited an enhanced UV photo-luminescent emission with respect to previously prepared non-fibrous homologues.

Frequently, the molecules employed for designing LMWGs contain one or more chiral centers and the gel can transcribe its chiral information to the nano-scale entity through the hierarchical self-assembly. Shao et al. [5] reported on the preparation and formation process of helical phenolic resins through a sol–gel transcription method. A pair of bola-type chiral LMWGs derived from valine were used as templates, while 2,4-dihydroxybenzoic acid and formaldehyde were used as precursors. The helical phenolic resins exhibited optical activity, showing that the chiral nature of the LMWGs was transcribed to the resin. Finally, a review presented by Hu and Yang [6] summarizes results reported in the last several years on the preparation of silica nanotubes with single-handed helices using chiral LMWGs derived from amino acids that self-assemble into helical fibers and twisted/coiled nanoribbons prepared through a sol–gel transcription process. 

To conclude, this special issue compiles original examples of gels used as templates for the syntheses of shape-controlled nanostructured materials and highlights the importance of this methodology and the versatility of gels, particularly LMWGs, as molecular templates for the preparation of well-defined and sometimes chiral nanostructured inorganic and organic materials. Although there are many known methods for the preparation of nanostructured materials, the use of gels as templates is still a promising and challenging approach. I really hope reading this special issue will be inspiring.
